# *Hypericum hircinum* L.: Botany, Traditional Uses, Phytochemistry, and Pharmacological Properties

**DOI:** 10.3390/plants14101451

**Published:** 2025-05-13

**Authors:** Noemi Tocci, Manuel Pramsohler, Lorenza Conterno, Tobias Weil

**Affiliations:** 1 Laimburg Research Centre, Pfatten/Vadena, 39040 Auer/Ora, Italy; manuel.pramsohler@laimburg.it (M.P.); lorenza.conterno@laimburg.it (L.C.); 2 Research and Innovation Centre, Fondazione Edmund Mach, 38098 San Michele all’Adige, Italy; tobias.weil@fmach.it

**Keywords:** *Hypericum hircinum*, *Hypericum*, traditional uses, phenols, essential oils, antimicrobial, pharmacology

## Abstract

*Hypericum hircinum* L., commonly known as goat St. John’s wort or stinking tutsan, is a medicinal plant native to the Mediterranean basin and widespread across Europe and parts of the Middle East. It has a long history of traditional uses in folk medicine to treat respiratory diseases, wounds, and burns and to relieve migraine, rheumatism, and muscular pains. Despite numerous scientific studies shading light on the phytochemical profile and on the beneficial properties of the plant extracts, a comprehensive overview of the current knowledge is missing. In this paper, we summarized the available information on botany, traditional uses, phytochemistry, and pharmacological properties of *Hypericum hircinum* from peer-reviewed articles published till March 2025 in PubMed, ScienceDirect, Wiley, Springer, ACS, Scielo, and Web of Science databases. The presence of numerous valuable compounds, including terpenes, phenolic acids, flavonoids, and phloroglucinols, is reported as well as the wide range of pharmacological properties, such as antimicrobial, antifungal, antiviral, antidepressant, anti-collagenase, anti-α-glucosidase, and antioxidant activities, together with non-pharmacological properties. The data reported in this review contribute to a deeper understanding of the biological properties of the species and pave the way for further investigation of its potential applications.

## 1. Introduction

The genus *Hypericum* (Hypericaceae family) includes more than 500 species distributed all over the world, with a center of species richness in Eurasia [1,2].

Plants belonging to the genus *Hypericum* are well known in the traditional medicine of several countries, where they are used for the treatment of pathological conditions such as mild depression, wounds and burns, diarrhea, pain, fevers, and poisoning from venomous animal bites [3]. The traditional Chinese medicine, one of the oldest and most well-structured, utilizes 64 species of *Hypericum*, including 33 endemic Chinese species, like *H. sampsonii* Hance, together with species more widely distributed like *Hypericum attenuatum* Fisch. ex Choisy, *Hypericum erectum* Thunb., *Hypericum japonicum* Thunb., and *Hypericum scabrum* L. [3,4].

*Hypericum* species are very popular as ailments also in the European folk medicine. The Mediterranean Basin, hosting more than 150 *Hypericum* taxa, is considered a hot spot of diversity for this genus [5]. Out of all the *Hypericum* species, *Hypericum perforatum* L. is one of the most studied plants in the world and is also considered the most important medicinal plant of the 20th century [6]. It is included in the European, Chinese, and Indian pharmacopoeias, and it is commonly used for the treatment of mild depression [2]. Numerous products based on *H. perforatum* L. extracts are available on the market, and to meet the demand for raw material, the species is cultivated on a large scale not only in Europe [7]. The species has also been the subject of biotechnological research mainly focused on the development of protocols for micropropagation and the production of secondary metabolites.

Many other Mediterranean species have been proven to be valuable sources of bioactive compounds for treating diverse pathological conditions with potential, even superior to those of *H. perforatum* L. [5,8]. The species include *H. androsaemum* L., showing diuretic, hepatoprotective, and antidepressant properties; *H. calycinum* L. with antioxidant and beneficial effects on the nervous system; and *H. origanifolium* Willd. with anti-aging and anxiolytic effects [9,10,11].

However, due to the broad spectrum of beneficial properties of the genus, the investigation of scarcely distributed or endemic species represents an unexplored, abundant, and reliable source of pharmacologically active metabolites for exploitation in the pharmaceutical and cosmeceutical industry. Among the scarcely distributed species, *H. hircinum* L., exclusively present in the Mediterranean basin, is an interesting medicinal plant attracting the interest of the scientific community, as evidenced by the increasing number of publications registered in the last 25 years.

The purpose of this review is to provide a comprehensive overview of the available knowledge on the botany, traditional uses, chemical profile, and pharmacological and non-pharmacological activity of *H. hircinum* L. based on scientific reports and evidence.

**Figure 1:**
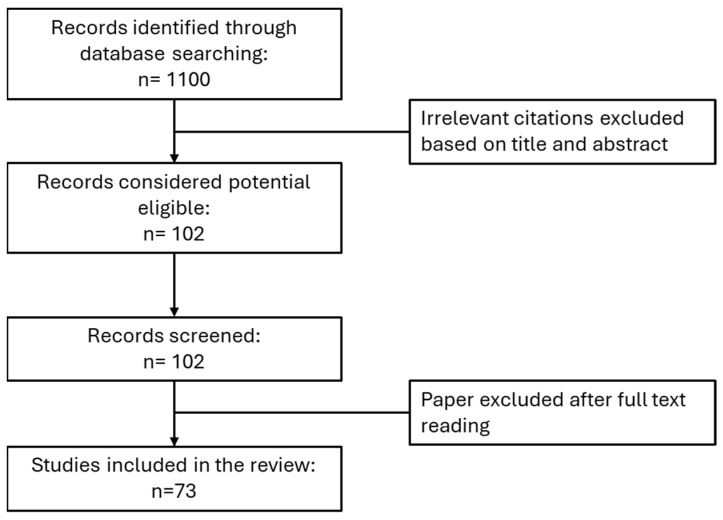
A flow chart of the literature selection process.

Through the review of 73 articles, chosen according to the process illustrated in Figure 1, a detailed chemical profile of essential oils and phenolic compounds has been made available, together with a description of traditional uses and pharmacological activities, providing valuable insight into the potential of *H. hircinum* for therapeutic use. Considerations on future challenges and investigations are also reported.

## 2. Botanical Overview

*H. hircinum* L. is a perennial glabrous shrub that can grow up to 3 m tall. It typically occurs in damp or shaded areas at altitudes between 300 and 1200 m. The species is tetraploid and contains 2n = 4x = 40 chromosomes [12,13]. Morphologically *H. hircinum* L. is characterized by erected to spreading or pendulous branches and four (young individuals)- or two-lined stems with internodes measuring 2 to 9 mm. The leaves are sessile to subsessile, are oval to lanceolate shaped, and show small glands. When crushed, they emit a characteristic goat-like odor, which is why the plant is also known as stinking tutsan or goat St. John’s wort. The inflorescences are apical, with a cylindrical–pyramidal shape, and consist of about 20 flowers. Flowers have a corolla formed by five golden yellow petals measuring 25 to 40 mm in diameter. They have long stamens measuring 12 to 22 mm. The fruits are ovoid to cylindrical capsules that are incompletely dehiscent, containing orange to reddish-brown seeds.

**Figure 2:**
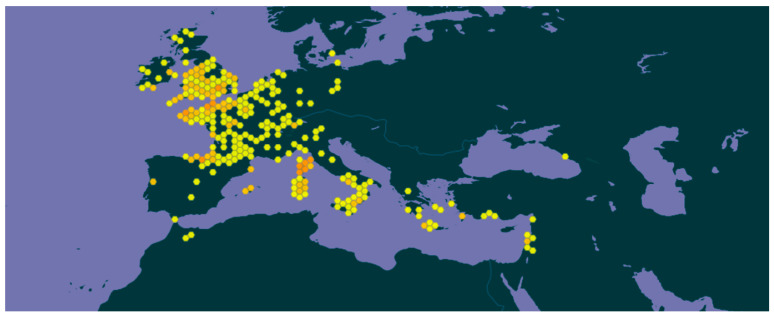
Distribution of *Hypericum hircinum* L. in Europe. Source: *H. hircinum* L. in GBIF Secretariat (2023). GBIF Backbone Taxonomy. The color scale reflects the frequency of observations. Checklist dataset https://doi.org/10.15468/39omei accessed via GBIF.org on 10 March 2025.

*H. hircinum* L. is distributed in the Mediterranean and the Middle East and is listed among the plants of the spontaneous flora of France, Spain, and Italy. Its actual distribution is extended to a wider area, including Greece, Portugal, Morocco, Turkey, Cyprus, Syria, Lebanon, Israel, Saudi Arabia, the British Isles, and the Balkans (Figure 2) [14,15].

According to the current classification, *H. hircinum* L. is taxonomically placed in the *Androsaemum* section and shows a high tendency to hybridize with similar taxa [5]. Based on morphological traits, five subspecies of *H. hircinum* L. can be distinguished [16]:

- *H. hircinum* subsp. *albimontanum* (Greuter) N. Robson found in Cyprus and some Greek Islands (Samos, Andros, Crete).
- *H. hircinum* subsp. *cambessedesii* (Nyman) Sauvage endemic to the Balearic Islands.
- *H. hircinum* subsp. *hircinum* native to Sardinia (Italy) and Corsica (France).
- *H. hircinum* subsp. *metroi* (Maire and Sauvage) Sauvage native to Morocco.
- *H. hircinum* subsp. *majus* (Aiton) N. Robson, widely distributed and often cultivated in gardens, regarded as the most primitive form of *H. hircinum* L., mainly occurring in Southern Italy (mostly in Sicily) and Crete.

To our knowledge there are no published scientific articles on the domestication or cultivation of *H. hircinum* L. However, the species is commonly sold in garden stores and is often cultivated in gardens as an ornamental plant.

## 3. Traditional Uses

The use of *H. hircinum* L. as a medicinal plant dates to Ancient Greece. The species is reported as a member of the Hippocratic medicinal flora of Kos Island, being one of the herbs on which Hippocrates’ healing practices were based [17].

In Italy, *H. hircinum* L. preparations are components of the traditional medicine of the Southern part of the Country. The plant has been employed as remedy for the treatment of cough and bronchitis in the regions of Sardinia [18,19] and Basilicata (both in South Italy), where it is referred to with the vernacular name of “erva da bronchite” (herb to treat bronchitis) [19]. In the Castelmazzano village, located in Dolomiti Lucane mountain area, the use of the species is very popular, and the plant is cultivated as a constant in many gardens. Here traditionally, the aerial parts are collected during summer, dried and then used during fall–winter for the preparation of a complex decoction, including the leaves, seeds, and fruits of other species like mallow (*Malva sylvestris* L.), chamomile (*Matricaria recutita* L.), figs (*Ficus carica* L.), jujube fruits (*Ziziphus jujuba* Mill.), barley (*Hordeum vulgare* L.) or oat (*Avena sativa* L.), mulberry (*Morus alba* L. and *M. nigra* L.), laurum (*Laurus nobilis* L.), orange (*Citrus sinensis* L.), almond (*Prunus dulcis* Mill.), and apple (*Malus domestica* Borkh.) [20].

**Figure 3:**
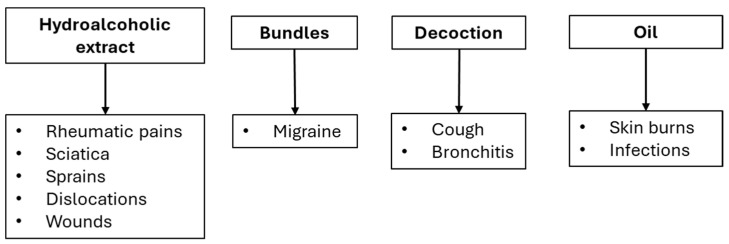
Schematic representation of preparation and uses of *H. hircinum* according to ethnopharmacological records.

In Sardinia, the oil of *H. hircinum* L. is also used to treat skin burns and infections, while the hydroalcoholic extract to relieve rheumatic pains, sciatica, sprains, and dislocations as well as for wound healing [19]. Finally, in Calabria (South Italy), bundles of *H. hircinum* L. are used as a remedy against migraines [21]. A schematic representation of traditional uses is shown in Figure 3.

## 4. Chemical Composition

The chemistry of the genus *Hypericum* has been largely studied, and numerous bioactive compounds like terpenoids, flavonoids, xanthones, naphthodianthrones, and phloroglucinols have been identified [22,23]. Several studies describing the chemical composition of both the essential oils (EO) and the phenolic profile of *H. hircinum* L. are available.

The most-used analytical method for the characterization of the chemical profile of *Hypericum* is chromatography. Volatile compounds are primarily identified by gas chromatography and gas chromatography coupled with mass spectrometry, while phenolic compounds are mainly detected and quantified by high-pressure and ultra-high-pressure liquid chromatography coupled with mass spectrometry [22].

### 4.1. Essential Oils

The *Hypericum* species are generally classified as plants with less oil concentration because of their low amount of EOs with yields < 1%, which in rare cases have been reported to be up to 3% and mainly accumulated in flowers and leaves [2,3,4,5,6,7,8,9,10,11,12,13,14,15,16,17,18,19,20,21,22,23,24]. The major constituents of *Hypericum* EOs are aliphatic hydrocarbons, sesquiterpenoids, and monoterpenes [2,24,25]. However, the oil composition has been found to be susceptible to environmental, physiological, and genetic factors, including seasonal fluctuations [24], phenological cycle [25], and geographic distribution [2].

*H. hircinum* L. EOs from both whole plants and individual organs have been characterized, and a list of compounds is reported in Table 1. In general, the EOs obtained from flowering aerial parts are rich in sesquiterpenes, monoterpenes, and alkanes [26,27]. More in details, EOs from flowering samples collected in Italy were characterized by cis-β-guajene (27.5%), δ-selinene (11.4%), n-nonane (10.2%), limonene (5.1%), β-pinene (4.3%), α-gurjunene (4.0%), and caryophyllene oxide (3.2%) as well as by the sesquiterpenes β-caryophyllene, α- and β-gurjunene, and caryophyllene oxide; by considerable amounts of 2,4-bis (1,1-dimethyl)-phenol in the β-glucosidase treated aqueous extract; and by the alkanes nonane, undecane, and 3-methylnonane [28] as major compounds, while EOs of plants from Greece and Lebanon have been found to be mainly composed of hydrogenated sesquiterpenes and monoterpenes (31.9% and 29.3% respectively), followed by oxygenated sesquiterpenes (18.6%) and monoterpene (15%), with (E)-caryophyllene and β-caryophyllene, caryophyllene oxide, and γ-terpinen being the most representative compounds [2,26] (Table 1).

Regarding EOs isolated from individual plant parts, α- and β-pinene, myrcene, and β-caryophyllene were identified as the main constituents of the leaf EOs in a population of *H. hircinum* L. from Turkey [2,29], while in plants collected in Southern Italy, nonane, α- and β-gurjunene, caryophyllene oxide, and β-caryophyllene showed the highest concentration [28].

Fruit EOs were found to be mainly composed of saturated aliphatic hydrocarbons and monoterpenes like nonane, undecane, α- and β-pinene, trans-pinocarveol, myrtenal, and α-terpineol [28].

These data appear different from those deriving from the analyses of leaf EOs of *H. hircinum* subps. *majus* (Aiton) N. Robson, investigated during different years, seasons, and phenological stages [30,31]. Here, the main compounds detected were cis-β-guaiene, isolongifolan-7-α-ol, (E)-caryophillene, δ-selinene, trans-Cadina-1(6), and 4-diene, while α- and β-gurjunene, β-pinene, and limonene were only detected in low or trace amounts.

EOs from fruits and flowers of *H. hircinum* subsp. *majus* (Aiton) N. Robson [30] contained, respectively, high amounts of monoterpenes, like limonene and β-pinene, and oxygenated monoterpenes and monoterpene hydrocarbons, with limonene, δ-selinene, isolongifolan-7-α-ol, β-pinene, limonene, α-terpineol, (E) caryophyllene, caryophyllene oxide, α-terpineol, nonane, and undecane being the most abundant (Table 1).

**Table 1:** Compounds found in essential oils extracted from both whole plants and individual organs of several *H. hircinum* L. subspecies.

Compound	Plant Part	Subspecies	Reference
(E,E)-α-farnesene	F L	*hircinum/majus*	[26,27,30,31]
(E)-caryophyllene	Fr L	*majus*	[30,31]
(E)-β-cymene	F L	*hircinum/majus*	[26,28,29,30,31]
(E)-β-farnesene	F L	*hircinum/majus*	[26,30,31]
(Z)-β-Ocimene	L	*hircinum/majus*	[28,29,30]
2-nonanone *	F Fr L	*hircinum*	[26,27]
3-methylnonane	F Fr L	*hircinum/majus*	[26,27,30,31]
9-epi-(E)-caryophyllene	L	*majus*	[30]
Acetophenone	Fr	*majus*	[30]
allo-aromadendrene	F L	*hircinum/majus*	[26,27,30]
Aromadendrene	F Fr L	*hircinum/majus*	[26,30]
Benzaldehyde	F	*majus*	[30]
Benzene acetaldehyde	F L	*hircinum/majus*	[26,30,31]
Benzyl benzoate	F Fr L	*majus*	[30,31]
Borneol	F Fr L	*hircinum/majus*	[26,27,30]
Camphene	F Fr L	*hircinum/majus*	[21,22,23,24,25,26,27,28,29,30]
Camphor	F Fr L	*hircinum*	[19,20,21]
Carvone	F Fr L	*hircinum/majus*	[26,27,30]
Caryophyllene oxide	F Fr L	*hircinum/majus*	[21,22,23,24,25,26,27,28,29,30]
cis-carveol	F Fr L	*hircinum/majus*	[26,30]
cis-verbenol	Fr	*majus*	[30]
cis-β-guaiene	F L	*hircinum/majus*	[26,30,31]
cis,trans-nepetalactone	F L	*hircinum*	[21]
Cumine aldehyde	Fr	*majus*	[30]
Cyclosativene	L	*majus*	[30]
Decanal	F Fr L	*hircinum/majus*	[26,30,31]
Decane	L	*majus*	[30]
Docosane	L	*majus*	[30,31]
Dodecane	L	*majus*	[30,31]
endo-Fenchol	F Fr	*majus*	[30]
epi-α-cadinol	Fr L	*majus*	[30]
Eucalyptol	F L	*hircinum*	[21]
Geraniol	F Fr L	*hircinum/majus*	[30,31]
Guaiazulene	F Fr L	*majus*	[30,31]
Heptacosane	F Fr L	*majus*	[30,31]
Hexacosane	L	*majus*	[30,31]
Hexadecane	F	*majus*	[30]
Hexadecanoic acid	F L	*majus*	[30,31]
Hexahydrofarnesyl acetone	F L	*hircinum*	[28]
Isoledene	F Fr L	*hircinum/majus*	[26,30,31]
Isolongifolan-7-α-ol	F Fr L	*majus*	[30,31]
Limonene	F Fr L	*hircinum/majus*	[26,27,28,29,30,31]
Linalool	F Fr L	*hircinum*	[26,27]
Linoleic acid	L	*majus*	[30,31]
Methyl dodecanoate	F Fr L	*hircinum/majus*	[26,30]
Myrcene	F Fr L	*hircinum/majus*	[27,28,29,30]
Myrtenal	Fr L	*hircinum/majus*	[26,27,30]
Myrthenol [27,30,31]	F Fr L	*hircinum/majus*	[26,30]
Nonacosane	F Fr L	*majus*	[30,31]
Nonanal	F Fr L	*hircinum/majus*	[26,30,31]
Nonane	F Fr L	*hircinum/majus*	[26,30,31]
Octane	F Fr L	*hircinum*	[26,27]
p-cymene	F Fr L	*hircinum/majus*	[27,30,31]
p-cymenene	Fr	*majus*	[30]
p-mentha-1,5-dien-8-ol	Fr	*majus*	[30]
Palustrol	F L	*hircinum*	[21]
Pentacosane	F Fr L	*majus*	[30,31]
Perilla alcohol	Fr	*majus*	[30]
Perilla aldehyde	Fr	*majus*	[30]
Phyllocladene	F L	*majus*	[30]
Phytol	F L	*majus*	[30,31]
Pinocarvone	F Fr L	*hircinum/majus*	[26,30,31]
Piperitone	Fr	*majus*	[30]
Selin-11-en-4-α-ol	Fr	*majus*	[30]
Spathulenol	F Fr L	*majus*	[30]
Terpinen-4-ol	F Fr L	*hircinum/majus*	[26,27,30]
Terpinolene	F Fr L	*hircinum/majus*	[26,27,28,29,30]
Tetracosane	F L	*majus*	[30,31]
Tetradecanoid acid	L	*majus*	[27]
trans-cadina-1, 4-diene	F Fr L	*hircinum/majus*	[26,27,28]
trans-carveol	F Fr L	*hircinum/majus*	[26,27,30]
trans-dihydrocarvone	Fr	*majus*	[30]
trans-limonene oxide	F	*majus*	[30]
trans-p-mentha-2,8-dien-1-ol	Fr	*majus*	[30]
trans-pinocarveol	F Fr L	*hircinum/majus*	[26,27,30]
trans-verbenolmajus	Fr	*majus*	[30]
Tricosane	F L	*majus*	[27,28]
Undecane	F Fr L	*hircinum/majus*	[27,28]
Valeranone	F L	*hircinum*	[27]
Verbenone	F Fr L	*hircinum/majus*	[26,27,30]
Viridiflorol	F Fr L	*hircinum/majus*	[26,30]
α-amorphene	F L	*hircinum*	[27]
α-campholenal	Fr	*majus*	[30]
α-campholene aldehyde *	Fr	*hircinum*	[26,27]
α-copaene	F Fr L	*hircinum/majus*	[26,30,31]
α-curcumene	F L	*hircinum*	[27]
α-eudesmol	F L	*hircinum*	[27]
α-gurjunene	F Fr L	*hircinum/majus*	[26,27,30,31]
α-himachalene	Fr	*majus*	[30]
α-humulene	F L	*hircinum/majus*	[26,28,29,30,31]
α -muurolene	F L	*hircinum*	[26]
α-pinene	F Fr L	*hircinum/majus*	[26,27,28,29,30,31]
α-selinene	F Fr L	*hircinum/majus*	[25,26]
α-terpinene	L	*hircinum*	[31]
α-terpineol	F Fr L	*hircinum/majus*	[26,27,28,29,30]
α-thujene	F Fr L	*majus*	[30]
β-bouronene *	F Fr L	*hircinum*	[26,27]
β-calacorene	F L	*hircinum/majus*	[26,30,31]
β-caryophyllene	F Fr L	*hircinum*	[27,28]
β-copaene	F L	*hircinum*	[27]
β-eudesmol	L	*majus*	[30,31]
β-fenchol	F Fr L	*hircinum*	[26,27]
β-gurjunene *	F Fr L	*hircinum*	[26,27]
β-maaliene	F L	*hircinum/majus*	[26,30,31]
β-myrcene	F L	*hircinum*	[26]
β-pinene	F Fr L	*hircinum/majus*	[26,27,28,29,30,31]
β-selinene	F Fr L	*hircinum/majus*	[26,30,31]
γ-cadinene	Fr	*hircinum/majus*	[27,30]
γ-elemene	F L	*hircinum*	[27]
γ-gurjunen	F L	*majus*	[30,31]
γ-muurolene	F L	*hircinum/majus*	[26,30,31]
γ-terpinene	F Fr L	*hircinum/majus*	[26,27,28,29,30]
δ-cadinene	F Fr L	*hircinum/majus*	[26,30,31]
δ-selinene	F Fr L	*hircinum/majus*	[26,30,31]
ρ-cymen-8-ol	F Fr	*majus*	[30]

* Tentative identification; F: flowers, Fr: fruits; L: leaves.

### 4.2. Phenolic Compounds

*Hypericum* species are sources of various bioactive phenolic compounds belonging to the polyketide metabolism and including phloroglucinols, naphtodianthrones, xanthones, flavonoids, and flavones. The distribution of these metabolites is of interest for the chemotaxonomy of the genus [1,32,33], and some of these compounds are regarded as chemotaxonomic markers suitable for resolving higher-order relationships within taxonomy (i.e., family to species) [1].

*H. hircinum* L., like all members of the *Androsaemum* section, accumulates phloroglucinols but not anthraquinones [7,34,35], even if traces of hypericin and pseudohypericin have been reported by Mandrone et al. and Odabaş et al. [36,37] in flowers of *H. hircinum* subsp. *majus* (Aiton) N. Robson. In a previous study by Tocci et al. [38], the phenolic profile of *H. hircinum* L. subsp. *majus* (Aiton) N. Robson was described and compared with other species collected in the same period and in the same geographical area in the northeastern Italy. As reported in Table 2, *H. hircinum* L. exhibited higher amounts of benzoate and cinnamates compared to *H. hirsutum* L., *H. maculatum* Crantz, and *H. perforatum* L. and the highest content of flavonols of all the species studied.

**Figure 4:**
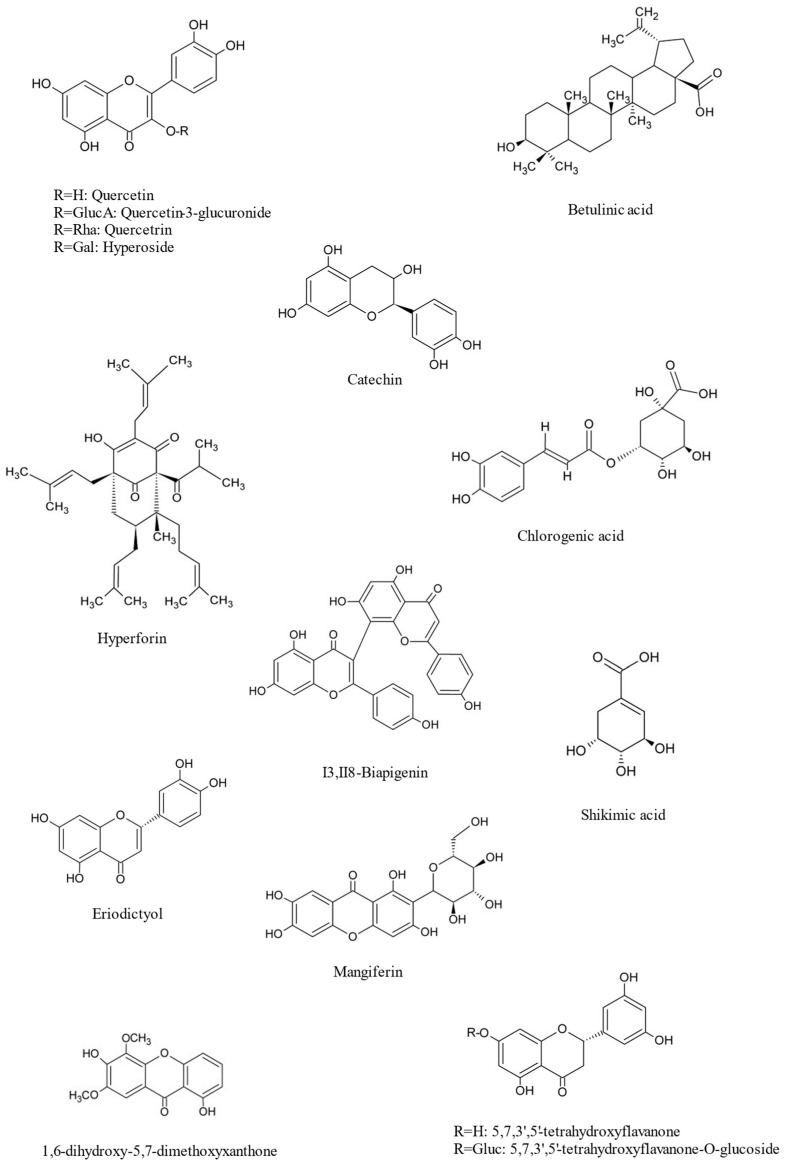
Chemical structures of major compounds in *H. hircinum* L. extracts.

Crude extracts of *H. hircinum* L. contain quercetin, quercetin-7-O-glucoside, quercitrin, eriodictyol, 1,6-dihydroxy-5,7-dimethoxyxanthone, (4R)-4-hydroxy-5,5-dimethyldihydrofuran-2-one, quercetin-3-O-β-D-glucopyranoside, eriodictyol-7-O-β-D-glucopyranoside, 5,7,3′,5-tetrahydroflavone, 5,7,3′,5-tetrahydroflavone-O-glucoside, and I3, II8-biapigenin and mangiferin, together with the chlorogenic and shikimic acids and the pentacyclic triterpenoid betulinic acid as major compounds [34,35,36,37,38,39,40,41] (Figure 4).

*H. hircinum* subsp. *majus* (Aiton) N. Robson is the best characterized subspecies. The ethanolic extract of the aerial parts of plants collected in Portugal were characterized by high amounts of hyperoside and quercitrin [42]. Extracts from plants collected in Central and Northern Italy confirmed hyperoside as the major compound and also reported consistent concentrations of chlorogenic acid, neochlorogenic acid, quercetin glycosides, isoquercitrin, quercitrin, and catechin and of the phloroglucinol hyperforin [38,43,44]. Plants collected in Sicily were characterized by the presence of 5-O-caffeoylquinic acid and cinnamic acids derivatives [34]. Odabaş et al. [37] analyzed the distribution of 17 phenolic compounds in leaves, stems, and flowers and found that flowers contained higher concentrations of amentoflavone, quercetin, catechin, and epicatechin in comparison with the other organs, while stem extracts were characterized by the lowest amounts of phenolic compounds.

## 5. Pharmacological Properties

**Figure 5:**
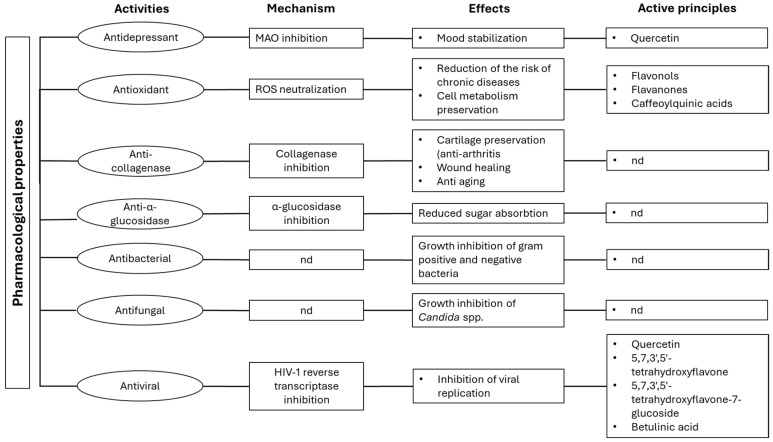
Pharmacological properties of *H. hircinum* L. extracts and EO. nd: not determined.

The genus *Hypericum* includes a large number of species with documented pharmacological properties, such as antidepressant, antimicrobial, anticancer, and wound healing [45,46]. Several properties have also been documented for *H. hircinum* L., including antimicrobial, antidepressive, antioxidant, and inhibition against matrix metallo-proteinase (MMP) activities, as reported in Figure 5.

### 5.1. Antibacterial Activity

Many species of the genus *Hypericum* are known for their beneficial effects against infectious diseases like skin inflammation and urinary and gastrointestinal disorders caused by microorganisms. The antibacterial activity of the genus has been extensively studied, and many reports underline its potential as source of valuable antimicrobial leads [47,48].

Despite the fact that essential oils are well known for their antimicrobial properties, the antibacterial properties of those deriving from *H. hircinum* L. have not been properly investigated so far. In the study of Pistelli et al. [39], the extracts of *H. hircinum* L., tested through the disc diffusion assay, showed growth inhibitory activity against the Gram-positive bacteria *Bacillus subtilis*, *Staphylococcus aureus*, and *Listeria monocytogenes* and against the Gram-negative bacteria *Salmonella enteritidis* and *Pseudomonas aeruginosa*. Through the same test, *H. hircinum* subps. *majus* (Aiton) N. Robson extracts revealed activity against *S. aureus* and *P. aeruginosa* [30]. Interestingly, chloroform extracts from *H. hircinum* subps. *majus* (Aiton) N. Robson and *H. hircinum* subsp. *albimontanum* (Greuter) N. Robson showed strong growth inhibition of multi-drug-resistant strains of *S. aureus* [43]. A study of Nogueira et al. [42], performed using the broth microdilution assay, demonstrated noteworthy properties against a panel of clinical drug-resistant strains of *Mycobacterium tuberculosis* and confirmed activity against *S. aureus*.

A study of Maggi et al. [30] documented activity of *H. hircinum* subsp. *majus* (Aiton) N. Robson oils against both negative (*E. coli*) and Gram-positive (*S. aureus*, *S. mutans*, and *B. subtilis*) bacteria; however, no defined minimum inhibitory concentration values are available.

### 5.2. Antifungal Activity

Fungal infections are considered a major health problem worldwide. The urgency of the pharmaceutical industry in searching for novel lead molecules for antifungal therapy has rekindled interest in plant derived natural products [6]. Here, an increasing number of publications highlights the antifungal potential of the genus *Hypericum* [32,38,49].

Representatives of the fungal genus *Candida*, such as *C. albicans*, are among the most prevalent and important causes of severe invasive infections, leading to high mortality and treatment costs [50]. Extracts and essential oils of *H. hircinum* subsp. *majus* (Aiton) N. Robson have shown activity against pathogenic *Candida* species [30,43]. Tocci et al. [38] described the strong anti-*Candida* properties of the extracts obtained from the infusion of the aerial parts of *H. hircinum* subsp. *majus* (Aiton) N. Robson in hot water, active against both fluconazole-resistant and -sensitive fungal strains, including *C. albicans*, and emerging pathogens like *C. parapsilosis* and *C. tropicalis* while showing no cytotoxicity to human cells in vitro. This study provided a scientific basis for the traditional use of teas based on *H. hircinum* L. to treat cough and bronchitis that are often exacerbated by fungal infections, particularly caused by *Aspergillus* and *Candida* spp. [51]. It suggested the species as a potential supplier of antifungal remedies and active principles, thus encouraging further investigations.

### 5.3. Antiviral Activity

Plants with antiviral properties have often been the object of investigations aiming to identify new lead compounds to inhibit virus replication. Despite the availability of more than 20 drugs currently approved for the treatment of the human immunodeficiency virus type-1 (HIV-1), HIV-1 still represents a worldwide health issue [52]. *Hypericum* spp. have been reported to show anti-HIV-1 properties, and this activity has been mainly attributed to the presence of different classes of compounds like hypericin and pseudohypericin [53] or phloroglucinols [54]. Ethanolic extracts obtained from *H. hircinum* L. were found to efficiently inhibit the HIV-1 reverse transcriptase-associated RNase H function, which is fundamental for viral replication in the host [41]. In addition to those of quercetin, 5,7,3′,5′-tetrahydroxyflavone, and 5,7,3′,5′-tetrahydroxyflavone-7-glucoside, the antiviral activity of the extract has been mainly attributed to the presence of betulinic acid (Figure 4), this latter known to inhibit viral replication, blocking viral entry and maturation in the host cells [52].

### 5.4. Effects on the Nervous System

The most famous species, *H. perforatum* L., is well known for its antidepressive activity and remedies for the treatment of mild-to-moderate depression are available in commerce. Also, other *Hypericum* species have been shown to exert antidepressant effects, both in vitro and in vivo tests, suggesting that the genus could be an excellent source of pharmaceuticals for the treatment of neurological disorders [55]. The proposed mechanism of action through which *Hypericum* extracts exert their beneficial effects is the inhibition of the enzymes monoaminoxidase (MAO) responsible for the inactivation of monoaminergic neurotransmitters. By avoiding the neurotransmitter breakdown, MAO inhibitors help in stabilizing the mood [40].

Methanolic extracts of *H. hircinum* L. showed in vitro inhibitory activity of MAO, and the active principle was identified as the flavonoid quercetin (Figure 1), which was found to be a selective inhibitor of MAO-A, an isomer responsible for the oxidative deamination of amines like norepinephrine, serotonin, and dopamin [40]. However, this effect was not observed in the in vivo Porsolt’s FST model of depressive states in rats using doses of quercetin ranging from 1 to 100 mg/kg. On the contrary, the open field test, an in vivo model used for measuring levels of anxiety, demonstrated an anxiogenic effect of the *H. hircinum* L. extract on mice, both at low and high concentrations [56].

### 5.5. Antioxidant Properties

Oxidative stress occurs when the detoxifying mechanisms of cells are not able to neutralize excessive reactive oxygen species (ROS) and free radicals that can cause important damage to cell metabolism. Oxidative stress in humans is involved in cell aging and in the development of serious vascular and neurological pathological states, like Alzheimer’s and Parkinson’s diseases, as well as to cancer. Antioxidants are playing a fundamental role to counteract detrimental effects of ROS, and phenolic compounds extracted from plants are important antioxidants. For instance, their assumption with the diet is considered essential for the prevention of the risk of strokes [57,58] and cancer [59]. Moreover, antioxidants are important supplements in the food industry for food preservation [60].

The antioxidant activity of extracts and fractions from *H. hircinum* L. were investigated in vitro using the ABTS, the DPPH, the FRAP-ferrozine (FRAP-FZ), and the β-carotene bleaching test [31,37,61]. This analysis revealed a rather high antioxidant activity of both ethanolic extract and decoction and highlighted the positive contribution of flavonol components, together with derivatives of flavanones and caffeoylquinic acids, to the antioxidant capacity of *H. hircinum* L. crude extract. The in vitro findings were also observed in vivo by a study by Shah et al. [62] demonstrating the protective antioxidant activity of *H. hircinum* L. extracts on cardiotoxicity that was artificially induced in rats by treatment with the antitumoral drug doxorubicin.

On the other hand, the antioxidant activity of *H. hircinum* subsp. *majus* (Aiton) N. Robson was further evaluated using the Rancimat and the DPPH radical scavenging methods, revealing only a moderate effect [44], highlighting the necessity for further in-depth investigations to better define the actual components, the extraction process, and the plant metabolism influencing the antioxidant properties.

### 5.6. Inhibitory Activity on Matrix Metallo-Proteinases (MMPs)

Matrix metallo-proteinases (MMPs) are a family of endoproteinases involved in the degradation of components of the extracellular matrix. MMPs are responsible for the degradation of cartilage associated with rheumatoid arthritis and osteoarthritis [63]; of newly formed extracellular matrix resulting in non-healing wounds [64,65]; and of elastin fibers in alveoli, playing an important role in the progression of chronic obstructive pulmonary disease [66]. The ethnopharmacological uses of *H. hircinum* L. as a remedy for rheumatic pains, wound healing, and bronchitis suggest a possible role as an MMP inhibitor. Extracts from *H. hircinum* L. have been shown to exert inhibitory activity against two members of the MMP family, collagenase [61] and α-glucosidase [36].

#### 5.6.1. Anti-Collagenase Activity

Collagens are the most abundant proteins in mammals and represent the main structural proteins in the extracellular matrix (ECM), where they play structural roles contributing to tensile strength in skin and to resistance to traction in ligaments [67].

Collagenases degrade collagen in smaller peptides and are involved in the maintenance of numerous healthy processes, including morphogenesis, tissue remodeling, and wound healing; in multiple pathologies, including tumor cell spreading (metastasis), arthritis, glomerulonephritis, periodontal disease, tissue ulcerations, cardiovascular disease, neurodegenerative diseases; and in skin aging [68]. Therefore, the search for collagenase inhibitors is of considerable interest both for pharmaceutical and cosmetic applications.

Due to their well-reported anti-ECM-degrading enzyme activity, *Hypericum* species represent a largely unexplored source of bioactive molecules that can be utilized to develop cosmetic formulations exhibiting anti-aging properties. Anti-collagenase activity has been reported for several *Hypericum* species, including *H. androsaemum* L., *H. ascyron* L., *H. calycinum* L., *H. confertum* Choisy, *H. origanifolium* Willd., and *H. perforatum* L. [61,69,70,71].

The hydroalcoholic total extracts of *H. hircinum* L. showed in vitro inhibitory effects through a non-competitive mechanism on collagenase activity (IC_50_ value of 156.0 µg/mL), which were higher in fractions containing quercetin, caffeoylquinic acids, and 5,7,3′,5′-tetrahydroxyflavanone (Figure 1) [61].

#### 5.6.2. Anti α-Glucosidase Activities

Glucosidases are a class of enzymes involved in the hydrolysis of glycosidic bonds in oligosaccharides and glycoconjugates, playing an important role in many biological processes. The research of glycosidase inhibitors finds application in the development of new therapeutic strategies for the treatment of numerous diseases such as diabetes, obesity, cancer, viral infections, and genetic disorders. Plants have long traditional uses in the treatment of metabolic disorders, and the genus *Hypericum* has been reported as an important source of anti-hyperglycemic agents. Amongst others, *H. perforatum* L., *H. asyncron* L., and *H. linarioides* Bosse have been shown to possess anti-α-glucosidase activity. Notably, the availability of glucose and related monosaccharides is decreased by the inhibition of α-glucosidases, resulting in low sugar levels and reduced absorption of after-meal glucose. Unfortunately, the currently available α-glucosidases often cause undesirable gastrointestinal side effects. For this reason, the search for alternative inhibitors of α-glucosidase is of importance in the development of novel therapeutic strategies for the treatment of metabolic disorders such as hyperglycemia, type II non-insulin-dependent diabetes mellitus, and obesity. In a study performed on three different *H. hircinum* L. extracts (70% ethanol, 50% methanol in phosphate buffer, and decoction), Madrone et al. [36] reported a relevant inhibition activity of α-glucosidase comparable to those of luteolin and acarbose, the most widely prescribed drug, encouraging further studies to evaluate the application of *H. hircinum* L. preparations for the development of food supplements to combat hyperglycemia.

## 6. Toxicity and Safety Assessment

Plant extracts consist of a complex of biologically active compounds. Therefore, assessing toxicity and herb–drug interaction prior to the proposal of an herb-based product is essential. The toxicity and safety of *H. perforatum* L., which is already used in commercial products, have been extensively studied. The most common cause of *Hypericum*-related drug interaction is the modulation of the Cytochrome P450 (CYP450) enzyme, resulting in the reduction of drug efficacy in therapy [72].

Currently, there are no available studies on the herb–drug interactions, safety, and toxicology of *H. hircinum* L. in vivo.

It has been demonstrated that the administration of *H. hircinum* L. extracts in mice reduces the cardiotoxicity of doxorubicin, a drug with potent and broad spectrum antitumor activity against a variety of human solid tumors and hematological malignancies [62].

Some in vitro cytotoxicity data are available. *H. hircinum* L. extracts showed toxicity at the highest dosages tested (500 µg/mL) on African green monkey kidney cells [36] and lower cytotoxicity than *H. perforatum* L. on the murine fibroblasts cell line, NIH/3T3 (LC_50_ 180 and 101 μg/mL, respectively, in dark condition and 140 and 33 μg/mL in light exposure) [72]. A low cytotoxicity was also reported to human fibroblasts for *H. hircinum* subps. *majus* (Aiton) N. Robson [38].

## 7. Non-Pharmacological Properties

### Allelopathic Activity

The extensive use of synthetic herbicides presents a significant threat for human and environmental health and has led to the search for biological alternatives for weed control. Plant extracts with allelopathic activity that can influence the germination and growth of other plants could represent valid alternatives to the use of synthetic herbicides. However, there is limited knowledge regarding the allelopathic potential of the genus *Hypericum*. In a screening project involving 17 Mediterranean plant species, the aqueous extract of *H. hircinum* subsp. *majus* (Aiton) N. Robson emerged as a promising candidate for weed biocontrol. In particular, the extract inhibited seed germination and exerted phytotoxic effects in pot cultures, suggesting its potential use as an herbicide [73]. In another study, essential oils from *H. hircinum* L. collected in Southern Italy showed slight inhibitory effects on the germination and radicle elongation in garden cress [27].

## 8. Materials and Methods

Peer-reviewed articles were consulted using the search terms “*Hypericum hircinum*”, “stinking tutsan”, and “Goat St John’s wort” in databases such as PubMed, ScienceDirect, Wiley, Springer, ACS, Scielo, Web of Science, and other web search tools like Google Scholar and Google Search. The research was performed applying several terms: “*Hypericum* AND *hircinum*”, “*Hypericum hircinum phytochemistry*”, “*Hypericum hircinum ethnopharmacology*”, “*Hypericum hircinum composition*”, “*Hypericum* AND *ethnopharmacology*”, and “*Hypericum hircinum activity*”. Exclusion criteria were applied for duplicates, and specific evaluation parameters were used to check whether the selected articles fulfilled the established criteria. All articles reviewed were published until March 2025 and are in English and Italian language.

## 9. Conclusions

*H. hircinum* L. is a Mediterranean plant used in the traditional medicine of Southern Italy to treat bronchitis, rheumatic and muscular pains, wound healing, skin infections, and migraines.

Evidence from the literature demonstrates that *H. hircinum* L. is rich in bioactive metabolites like terpenes, alkanes, phloroglucinols, flavonoids, xanthones, and phenolic acids.

Although many chemical components have been identified, only a few pure compounds have been tested for their biological activity to clarify the pharmacological mechanism of action and the metabolites responsible for the activities. In this context, the limited distribution of the species could represent a limit for the retrieval of the raw material. The application of biotechnological production processes for the in vitro propagation of the species and for the study of secondary metabolism could represent a successful tool.

*H. hircinum* L. has been shown to exert various pharmacological properties, including antimicrobial properties, antioxidant properties, antidepressant properties, and the inhibition of matrix metallo-proteinases, supporting the traditional uses of the plant.

Despite all the encouraging data, most of the studies were only performed in vitro, and in vivo models to provide scientific evidence are still missing. A deeper investigation of *H. hircinum* L. to better exploit the potentiality of the plant is needed. The investigation of toxicity and safety assessment especially represents a crucial challenge before delving into in vivo studies.

## Figures and Tables

**Table 2:** Phenolic profile of *H. hircinum* subps. *majus* (Aiton) N. Robson in comparison with other species.

*	*H. hircinum* subsp. *majus*	*H. hirsutum*	*H. maculatum*	*H. montanum*	*H. perforatum*
	µg/g Extract				
Benzoate and cinnamates	427 ± 47.1	45.90 ± 3.65	243.22 ± 1.14	390.72 ±6.17	112.60 ± 10.50
Flavonols	9393.64 ± 50.3	7470.05 ± 28.01	6555.52 ± 30.21	5284.31 ± 13.25	7189.19 ± 47.26
Flavones	0.65 ± 0.036	2.22 ± 0.02	1.18 ± 0.01	3.04 ± 0.01	0.45 ± 0.01
Flavanones	0.44 ± 0.01	1.43 ± 0.03	0.58 ± 0.02	2.30 ± 0.01	0.82 ± 0.02
Chalcones	0.11 ± 0.01	0.42 ± 0.01	0.32 ± 0.01	nd	0.31 ± 0.01
Coumarines	0.23 ± 0.01	0.07 ± 0.00	0.14 ± 0.00	0.55 ± 0.01	0.22 ± 0.00
Flavan-3-ols	53.12 ± 1.24	74.12 ± 3.12	100.09 ± 4.81	58.28 ± 1.53	59.47 ± 2.47
Stilbenoids	0.06 ± 00	0.91 ± 0.02	2.24 ± 0.00	0.99 ± 0.01	3.32 ± 0.05
Phloroglucinols	nd	nd	nd	20.00 ± 1.91	8050 ± 47.1565
Naphtodianthrones	nd	30.00 ± 1.79	20.00 ± 1.71	40.00 ± 2.67	150.00 ± 25.10

* From Tocci et al. [38]. nd: not detected.

## Data Availability

No new data were created to write this paper.

## References

[1] Crockett S.L., Robson N.K. (2011). Taxonomy and chemotaxonomy of the genus *Hypericum*. Med. Aromat. Plant Sci. Biotechnol..

[2] Grafakou M.E., Barda C., Karikas G.A., Skaltsa H. (2022). *Hypericum* essential oils—Composition and bioactivities: An update (2012–2022). Molecules.

[3] Caldeira G.I., Gouveia L.P., Serrano R., Silva O.D. (2022). *Hypericum* genus as a natural source for biologically active compounds. Plants.

[4] Zhang R., Ji Y., Zhang X., Kennelly E.J., Long C. (2020). Ethnopharmacology of Hypericum species in China: A comprehensive review on ethnobotany, phytochemistry and pharmacology. J. Ethnopharmacol..

[5] Nürk N.M., Blattner F.R. (2010). Cladistic analysis of morphological characters in Hypericum (Hypericaceae). Taxon.

[6] Gurib-Fakim A. (2006). Medicinal plants: Traditions of yesterday and drugs of tomorrow. Mol. Asp. Med..

[7] Kwiecień I., Nicosia N., Ekiert H. (2021). Cultivation of *Hypericum perforatum* (St. John’s Wort) and biotechnological approaches for improvement of plant raw material quality. Medicinal Plants: Domestication, Biotechnology and Regional Importance.

[8] Stojanovic G., Dordevic A., Smelcerovic A. (2013). Do other *Hypericum* species have medical potential as St. John’s wort (*Hypericum perforatum*)?. Curr. Med. Chem..

[9] Ersoy E., Ozkan E.E., Boga M., Mat A. (2020). Evaluation of in vitro biological activities of three *Hypericum* species (*H. calycinum, H. confertum*, and *H. perforatum*) from Turkey. S. Afr. J. Bot..

[10] Yaşar Ş.N., Can Ö.D., Öztürk N., Sagratini G., Ricciutelli M., Vittori S., Maggi F. (2013). Central nervous system activities of *Hypericum origanifolium* extract via GABAergic and opioidergic mechanisms. Phytother. Res..

[11] Boran R. (2018). Investigations of anti-aging potential of *Hypericum origanifolium* Willd. for skincare formulations. Ind. Crops Prod..

[12] Bruňáková K., Bálintová M., Henzelyová J., Kolarčik V., Kimáková A., Petijová L., Čellárová E. (2021). Phytochemical profiling of several *Hypericum* species identified using genetic markers. Phytochemistry.

[13] Butiuc-Keul A., Coste A., Budahn H., Dunemann F., Farcas A., Postolache D., Klocke E. (2022). Analysis of Hypericum accessions by DNA fingerprinting and flow cytometry. Acta Bot. Croat..

[14] Euro+Med PlantBase—The Information Resource for Euro-Mediterranean Plant Diversity. Published on the Internet The Euro+Med Plantbase Project. https://ww2.bgbm.org/EuroPlusMed/query.asp.

[15] GBIF|Global Biodiversity Information Facility. https://www.gbif.org/.

[16] Robson N.K.B. (1985). Studies in the genus *Hypericum* L. (Guttiferae). 3. Sections 1. Campylosporus to 6a. Umbraculoides. Bull. Brit. Mus. (Nat. Hist.) Bot..

[17] Fanouriou E., Kalivas D., Daferera D., Tarantilis P., Trigas P., Vahamidis P., Economou G. (2018). Hippocratic medicinal flora on the Greek Island of Kos: Spatial distribution, assessment of soil conditions, essential oil content and chemotype analysis. J. Appl. Res. Med. Aromat. Plants.

[18] Atzei A.D. (2003). Le piante nella tradizione popolare della Sardegna.

[19] Ballero M., Floris R., Poli F. (1997). Le piante utilizzate nella medicina popolare nel territorio di Laconi (Sardegna Centrale). Boll. Soc. Sarda Sci. Nat..

[20] Pieroni A., Quave L.C., Santoro R.F. (2004). Folk pharmaceutical knowledge in the territory of the Dolomiti Lucane, inland Southern Italy. J. Ethnopharmacol..

[21] Mazzei R., De Marco E.V., Gallo O., Tagarelli G. (2018). Italian folk plant-based remedies to heal headache (XIX–XX century). J. Ethnopharmacol..

[22] Ion V., Ielciu I., Cârje A.G., Muntean D.L., Crişan G., Păltinean R. (2022). *Hypericum* spp.—An Overview of the Extraction Methods and Analysis of Compounds. Separations.

[23] Tanaka N., Kashiwada Y. (2021). Characteristic metabolites of *Hypericum* plants: Their chemical structures and biological activities. J. Nat. Med..

[24] Guedes A.P., Franklin G., Fernandes-Ferreira M. (2012). *Hypericum* sp.: Essential oil composition and biological activities. Phytochem. Rev..

[25] Schwob I., Bessiere J.M., Masotti V., Viano J. (2004). Changes in essential oil composition in Saint John’s wort (*Hypericum perforatum* L.) aerial parts during its phenological cycle. Biochem. Syst. Ecol..

[26] Hani N., Hatem N., Safa B. (2023). Chemical Composition of Essential Oils of Different Hypericum Species Growing Wild in Shouf Biosphere Reserve, Lebanon. Jpn. J. Med. Res..

[27] Marandino A., Martino L.D., Mancini E., Milella L., Feo V.D. (2011). Chemical composition and possible in vitro anti-gemination activity of three *Hypericum* essential oils. Nat. Prod. Commun..

[28] Bertoli A., Pistelli L., Morelli I., Spinelli G., Menichini F. (2000). Constituents of *Hypericum hircinum* oils. J. Essent. Oil Res..

[29] Kiyan H.T., Demmirci B., Can Baser K.H., Demerici F. (2014). The in vivo evaluation of anti-angiogenic effects of *Hypericum* essential oils using the chorioallantoic membrane assay. Pharm. Biol..

[30] Maggi F., Cecchini C., Cresci A., Coman M.M., Trillini B., Sagratini G., Papa F., Vittori S. (2010). Chemical composition and antimicrobial activity of *Hypericum hircinum* subsp. *majus* essential oil. Chem. Nat. Compd..

[31] Quassinti L., Lupidi G., Maggi F., Sagratini G., Papa F., Vittori S., Bianco A., Bramucci M. (2013). Antioxidant and antiproliferative activity of *Hypericum hircinum* L. subsp. *majus* (Aiton) N. Robson essential oil. Nat. Prod. Res..

[32] Tocci N., Perenzoni D., Iamonico D., Fava F., Weil T., Mattivi F. (2018). Extracts from *Hypericum hircinum* subsp. *majus* Exert Antifungal Activity Against a Panel of Sensitive and Drug-Resistant Clinical Strains. Front. Pharm..

[33] Tocci N., Weil T., Perenzoni D., Narduzzi L., Madriñán S., Crockett S., Nürk N.M., Cavalieri D., Mattivi F. (2018). Phenolic profile, chemical relationship and antifungal activity of Andean *Hypericum* species. Ind. Crops Prod..

[34] Cirak C., Seyis F. (2023). Phenolic constituents of six *Hypericum* species from Türkiye and their chemotaxonomic relevance. S. Afr. J. Bot..

[35] Lazzara S., Carrubba A., Napoli E. (2020). Variability of hypericins and hyperforin in *Hypericum* species from the Sicilian flora. Chem. Biodivers..

[36] Giovino A., Carrubba A., Lazzara S., Napoli E., Domina G. (2020). An integrated approach to the study of *Hypericum* occurring in Sicily. Turk. J. Bot..

[37] Mandrone M., Scognamiglio M., Fiorentino A., Sanna C., Cornioli L., Antognoni F., Poli F. (2017). Phytochemical profile and α-glucosidase inhibitory activity of Sardinian *Hypericum scruglii* and *Hypericum hircinum*. Fitoterapia.

[38] Odabaş M., Radusiene J., Ivanauskas L., Jakstas V., Camas N., Kayikci S. (2016). Secondary metabolites in *Hypericum* species and their distribution in different plant parts Jonažolių rūšių antriniai metabolitai ir jų pasiskirstymas augalų dalyse. Zemdirbyste.

[39] Pistelli L., Bertoli A., Zucconelli S., Morelli I., Panizzi L., Menichini F. (2000). Antimicrobial activity of crude extracts and pure compounds of *Hypericum hircinum*. Fitoterapia.

[40] Chimenti F., Cottiglia F., Bonsignore L., Casu L., Casu M., Floris C., Secci D., Bolasco A., Chimenti P., Granese A. (2006). Quercetin as the Active Principle of *Hypericum hircinum* exerts a selective inhibitory activity against MAO-A: Extraction, biological analysis, and computational study. J. Nat. Prod..

[41] Esposito F., Sanna C., Del Vecchio C., Cannas V., Venditti A., Corona A., Bianco A., Serrilli A.M., Guarcini L., Paroli C. (2013). *Hypericum hircinum* L. Components as new single-molecule inhibitors of both HIV-1 reverse transcriptase-associated DNA polymerase and ribonuclease H activities. Pathog. Dis..

[42] Nogueira T., Medeiros M.A., Marcelo-Curto M.J., Garcia-Perez B.E., Luna-Herrera J., Costa M.C. (2013). Profile of antimicrobial potential of fifteen *Hypericum* species from Portugal. Ind. Crops Prod..

[43] Cecchini C., Cresci A., Coman M.M., Ricciutelli M.M., Sagratini G., Vittori S., Lucarini D., Maggi F. (2007). Antimicrobial activity of seven *Hypericum* entities from Central Italy. Planta Med..

[44] Sagratini G., Ricciuitelli M., Vittori S., Ozturk N., Ozturk Y., Maggi F. (2008). Phytochemical and antioxidant analysis of eight *Hypericum* taxa from Central Italy. Fitoterapia.

[45] Xiao C.Y., Mu Q., Gibbons S. (2020). The phytochemistry and pharmacology of *Hypericum*. Prog. Chem. Org. Nat. Prod..

[46] Vincent O.M., Nguta J.M., Mitema E.S., Musila F.M., Nyak D.M., Mohammed A.H., Gervason M.A. (2021). Ethnopharmacology, pharmacological activities, and chemistry of the *Hypericum* genus. J. Phytopharmacol..

[47] Gibbons S., Ohlendorf B., Johnsen I. (2002). The genus *Hypericum*-a valuable resource of anti-*Staphylococcal* leads. Fitoterapia.

[48] Dall’Agnol R., Ferraz A., Bernardi A.P., Albring D., Nör C., Sarmento L., Lamb L., Hass M., von Poser G., Schapoval E.E.S. (2003). Antimicrobial activity of some *Hypericum* species. Phytomedicine.

[49] Tocci N., Weil T., Perenzoni D., Moretto M., Nürk N.M., Madriñán S., Ferrazza R., Guella G., Mattivi F. (2020). Potent Antifungal Properties of Dimeric Acylphloroglucinols from *Hypericum mexicanum* and Mechanism of Action of a Highly Active 3′ Prenyl Uliginosin B. Metabolites.

[50] Rizzetto L., Weil T., Cavalieri D. (2015). Systems level dissection of *Candida* recognition by dectins: A matter of fungal morphology and site of infection. Pathogens.

[51] Ozyigit L.P., Monteiro W., Rick E.M., Satchwell J., Pashley C.H., Wardlaw A.J. (2018). Fungal bronchitis is a distinct clinical entity which is responsive to antifungal therapy. Chronic Respir. Dis..

[52] Aiken C., Chen C.H. (2005). Betulinic acid derivatives as HIV-1 antivirals. Trends Mol. Med..

[53] Gulick R.M., McAuliffe V., Holden-Wiltse J., Crumpacker C., Liebes L., Stein D.S., Meehan P., Hussey S., Forcht J., Valentine F.T. (1999). and AIDS Clinical Trials Group 150 and 258 Protocol Teams. Phase I studies of hypericin, the active compound in St. John’s wort, as an antiretroviral agent in HIV-infected adults: AIDS clinical trials group protocols 150 and 258. Ann. Intern. Med..

[54] Li D., Zhu H., Qi C., Xue Y., Yao G., Luo Z., Wang J., Zhang J., Du G., Zhang Y. (2015). Two new adamantyl-like polyprenylated acylphloroglucinols from *Hypericum attenuatum* choisy. Tetrahedron Lett..

[55] Saroya A.S., Singh J., Saroya A.S., Singh J. (2018). Neuropharmacology of Genus Hypericum: Hypericin and Hyperforin. Pharmacotherapeutic Potential of Natural Products in Neurological Disorders.

[56] Diana G., Capasso A., Quaranta E., De Feo V. (2007). Differential effects of three species of *Hypericum* in an open field test. Phytother. Res..

[57] Rautiainen S., Levitan E.B., Mittleman M.A., Wolk A. (2013). Total antioxidant capacity of diet and risk of heart failure: A population-based prospective cohort of women. Am. J. Med..

[58] Colarusso L., Serafini M., Lagerros Y.T., Nyren O., La Vecchia C., Rossi M., Bellocco R. (2017). Dietary antioxidant capacity and risk for stroke in a prospective cohort study of Swedish men and women. Nutrition.

[59] Zamora-Ros R., Fedirko V., Trichopoulou A., González C.A., Bamia C., Trepo E., Overvad K. (2013). Dietary flavonoid, lignan and antioxidant capacity and risk of hepatocellular carcinoma in the European prospective investigation into cancer and nutrition study. Int. J. Cancer.

[60] Faleiro M.L., Miguel G. (2020). Antimicrobial and antioxidant activities of natural compounds: Enhance the safety and quality of food. Foods.

[61] Mandrone M., Lorenzi B., Venditti A., Guarcini L., Bianco A., Sanna C., Ballero M., Poli F., Antognoni F. (2015). Antioxidant and anti-collagenase activity of *Hypericum hircinum* L.. Ind. Crops Prod..

[62] Shah S., Mohan M., Kasture S., Ballero M., Maxia A., Sanna C. (2013). Protective effect of *Hypericum hircinum* on doxorubicin-induced cardiotoxicity in rats. Nat. Prod. Res..

[63] Elliott S., Cawston T. (2001). The clinical potential of matrix metalloproteinase inhibitors in the rheumatic disorders. Drugs Aging.

[64] Schultz G.S., Ladwig G., Wysocki A. (2005). Extracellular matrix: Review of its roles in acute and chronic wounds. Worldw. Wounds.

[65] Vaalamo M., Weckroth M., Puolakkainen P., Kere J., Saarinen P., Lauharanta J., Saarialho-Kere U.K. (1996). Patterns of matrix metalloproteinase and TIMP-1 expression in chronic and normally healing human cutaneous wounds. Brit. J. Dermatol..

[66] Lee H.S., Kim W.J. (2022). The Role of Matrix Metalloproteinase in Inflammation with a Focus on Infectious Diseases. Int. J. Mol. Sci..

[67] Ricard-Blum S. (2020). Extracellular matrix networks: From connections to functions. Extracellular Matrix Omics.

[68] Amar S., Smith L., Fields G.B. (2017). Matrix metalloproteinase collagenolysis in health and disease. (BBA)-Mol. Cell Res..

[69] Antognoni F., Lianza M., Poli F., Buccioni M., Santinelli C., Caprioli G., Iannarelli R., Lupidi G., Damiani E., Beghelli D. (2017). Polar extracts from the berry-like fruits of *Hypericum androsaemum* L. as a promising ingredient in skin care formulations. J. Ethnopharmacol..

[70] Ersoy E., Ozkan E.E., Boga M., Yilmaz M.A., Mat A. (2019). Anti-aging potential and anti-tyrosinase activity of three Hypericum species with focus on phytochemical composition by LC–MS/MS. Ind. Crops Prod..

[71] Suryawanshi M.V., Gujarathi P.P., Mulla T., Bagban I. (2024). *Hypericum perforatum*: A comprehensive review on pharmacognosy, preclinical studies, putative molecular mechanism, and clinical studies in neurodegenerative diseases. Naunyn-Schmiedeberg’s Arch. Pharmacol..

[72] Napoli E., Siracusa L., Ruberto G., Carrubba A., Lazzara S., Speciale A., Cristani M. (2018). Phytochemical profiles, phototoxic and antioxidant properties of eleven Hypericum species–A comparative study. Phytochemistry.

[73] Araniti F., Sorgonà A., Lupini A., Abenavoli M.R. (2012). Screening of Mediterranean wild plant species for allelopathic activity and their use as bio-herbicides. Allelopath. J..

